# HAS THE AHLBÄCK CLASSIFICATION BEEN ACCURATELY DESCRIBED AND CITED?

**DOI:** 10.1590/1413-785220253306e296115

**Published:** 2025-11-10

**Authors:** Julio Cesar Gali, Igor Silva de Novais, Leonardo Altieri Carletti, Pedro Rinaldi Alves Cruz, Edie Benedito Caetano

**Affiliations:** 1 Pontificia Universidade Catolica de Sao Paulo, Faculdade de Ciencias Medicas e da Saude, Departamento de Cirurgia, Sao Paulo, SP, Brazil.; 2 Pontificia Universidade Catolica de Sao Paulo, Faculdade de Ciencias Medicas e da Saude, Sao Paulo, SP, Brazil.

**Keywords:** Knee, Osteoarthritis, Ahlbäck Classification, Joelho, Osteoartrose, Classificação De Ahlbäck, Osteoartrite

## Abstract

The classification of knee osteoarthritis allows assessment of disease severity and may be useful in guiding treatment decisions. One of the most widely used systems for this purpose is the Ahlbäck classification. This study aimed to compare the original description of the Ahlbäck classification with how it has been reported and cited by other authors in subsequent publications. We conducted a search in the PubMed, Embase, and Cochrane databases for articles containing exclusively the terms "knee", "osteoarthritis", "osteoarthrosis", and "Ahlbäck classification". After applying the inclusion and exclusion criteria, 64 articles remained. These articles were analyzed in two aspects: the description of the Ahlbäck classification (categorized as correct, partially correct, or incorrect) and the accuracy of the reference citation (correctly or incorrectly cited). Only 10 articles (15.6%) correctly described the Ahlbäck classification and cited the original source properly. In contrast, 37 publications (58.4%) contained errors both in the description of the classification and the citation. Conversely, 37 publications (58.4%) contained errors both in the description of the classification and in the bibliographic reference. The proportion of articles that accurately described and cited the Ahlbäck classification was markedly low, comprising only 15.6% of those included in this systematic review. ***Level of Evidence III; Systematic Review***.

## RESUMO

A classificação da gonartrose permite avaliar a gravidade da doença e pode ser útil na escolha do tratamento. Uma das classificações mais frequentemente usadas para esse propósito é a classificação de Ahlbäck. O objetivo deste estudo foi comparar a descrição original da classificação de Ahlbäck com o que outros autores escreveram sobre essa categorização e como a citaram, em artigos subsequentes. Realizamos uma pesquisa no PubMed, Embase e Cochrane por artigos contendo exclusivamente os conjuntos de palavras "joelho", "osteoartrite", "osteoartrose", "classificação de Ahlbäck". Após aplicarmos os critérios de exclusão e inclusão restaram 64 artigos. Esses artigos foram classificados em dois aspectos: quanto à descrição da classificação de Ahlbäck (correta, total ou parcial, e incorreta) e quanto às referências bibliográficas (citadas corretamente ou de forma equivocada). Apenas 10 artigos incluídos em nossa revisão sistemática (15,6%) descreveram corretamente a classificação de Ahlbäck e sua respectiva citação. Em contrapartida, 37 publicações (58,4%) apresentaram erros tanto na descrição da classificação quanto na referência bibliográfica. A porcentagem de artigos que descreveram corretamente tanto a escala de Ahlbäck quanto sua citação nas referências foi bastante reduzida, correspondendo a apenas 15,6% das publicações incluídas nesta revisão sistemática. ***Nível de Evidência III; Revisão Sistemática***.

## INTRODUCTION

The classifications of knee osteoarthritis aim primarily to reflect cartilage loss and disease severity, as well as to assist orthopedic surgeons in treatment selection, particularly for patients requiring surgery, such as total knee arthroplasty.^1^ In gonarthrosis, radiography is essential for assessing joint involvement and guiding treatment. Due to the varied presentations of knee osteoarthritis, clinical and radiographic classification is fundamental for defining management strategies and analyzing therapeutic outcomes.

**Figure 1:**
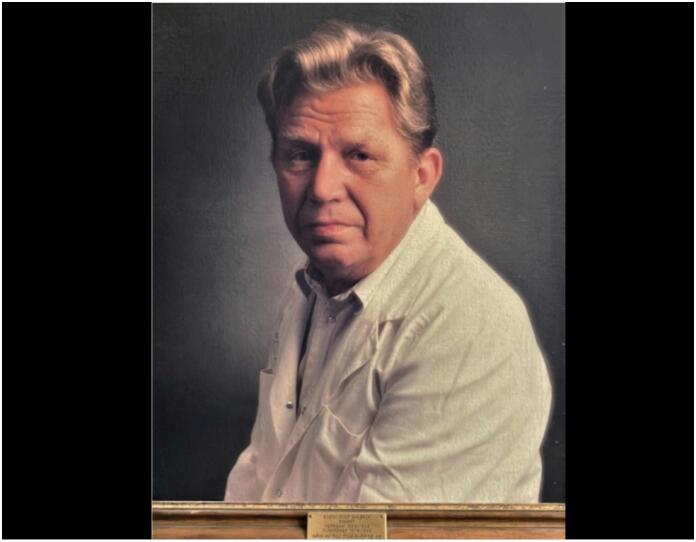
Portrait of Sven Olof Ahlbäck.

Source: St. Göran Hospital, Stockholm, Sweden.

In 1968, the Swedish radiologist Sven Olof Ahlbäck (1927–1995; Figure 1), from the Department of Radiology at St. Göran Hospital in Stockholm, published a monograph^2^ emphasizing the importance of weight-bearing anteroposterior knee radiographs to identify osteoarthritis in joints that appeared normal when assessed by other methods.

In this publication,^2^ Ahlbäck described the presence of bone defects, possibly caused by friction between the articular surfaces, classifying them into three categories according to size: less than 5 mm, between 5 and 10 mm, and greater than 10 mm. He also mentioned that osteoarthritis could be classified based on the location of cartilage destruction: medial femorotibial, lateral femorotibial, and patellofemoral.

In 1980, Ahlbäck et al.^3^ described a radiographic classification of knee osteoarthritis based on the measurement of articular cartilage and subchondral bone destruction. This analysis included 359 radiographs of knees with medial osteoarthritis, surgically treated with total arthroplasty. The description of this grading system is presented in Table 1.

**Table 1:** Ahlbäck & Rydberg Classification (based on articular cartilage and subchondral bone destruction).

Grade 1	Moderate cartilage destruction (joint space narrowing)
Grade 2	Complete cartilage destruction (joint space obliterated or nearly obliterated)
Grade 3	Minor bone attrition (<0.5 cm)
Grade 4	Moderate bone attrition (between 0.5 and 1.5 cm)
Grade 5	Severe bone attrition (>1.5 cm)

The "so-called Ahlbäck classification"^4^ was first cited in the scientific literature in 1987 by Lindberg et al.^5^ and continues to be used today. Although some authors have pointed out advantages in using this method,^1,6,7^ others have reported limitations such as low reliability^8^ or reproducibility^4^, as well as moderate interobserver reliability and moderate correlation with arthroscopic findings.^9^ Despite these limitations, the Ahlbäck classification is one of the most frequently cited in the literature^8,10^ and is commonly used to guide therapeutic decisions,^4,11^ including being recommended by the Knee Committee of the International Society of Arthroscopy, Knee Surgery, and Orthopaedic Sports Medicine (ISAKOS) for surgical indication of knee osteoarthritis.^12^

However, we identified publications that incorrectly described the Ahlbäck classifications and also cited their references inaccurately. Thus, our objective was to compare the classifications described in Ahlbäck's monograph^2^ and in the publication by Ahlbäck et al.^3^ with those mentioned in the articles that used them in their bibliography. We also sought to analyze how these classifications were cited, aiming to guide future authors who intend to use and reference them appropriately. Our goal, however, was not to assess the effectiveness of this classification or to compare it with other scales that also serve to categorize knee osteoarthritis.

## MATERIALS AND METHODS

This systematic review was conducted in accordance with the PRISMA 2020 statement guidelines.^13^ The search for articles that used the Ahlbäck classification^3^ was carried out by two independent authors (ISN and LAC) in the PubMed, Embase, and Cochrane databases, covering the period from 1987 — the date of the first publication that applied this grading — up to January 2, 2025. The following keywords were used: "knee," "osteoarthritis," "arthrosis," "Ahlbäck classification." For practical purposes, we considered both the Ahlbäck^2^ and the Ahlbäck et al.^3^ classifications as belonging to the "Ahlbäck classification," as generally described in the literature.^4^ The bibliographic search was manually filtered by the senior author (JCG) to identify eligible and ineligible articles. This selection was performed once and subsequently re-verified on two additional occasions.

Inclusion criteria were: full-text published articles; those in which the Ahlbäck classification was at least partially described in the text; and where the scales were listed in the references, in addition to articles published in English (with the exception of Ahlbäck et al.^3^, published in Swedish). Exclusion criteria were: publications that could not be fully retrieved from libraries or through requests to the authors by email; articles published in languages other than English; conference proceedings or abstracts; publications addressing subjects other than osteoarthritis; articles in which the Ahlbäck classifications were not found in the references; and publications in which the Ahlbäck categorizations were not presented in whole or in part, or where only a citation was provided.

The included articles were compared with the original descriptions of the classifications found in Ahlbäck's monograph^2^ and in the publication by Ahlbäck et al.^3^. We verified how these classifications were described and cited in the analyzed publications. The articles were organized by the senior author (JCG) regarding the description of the Ahlbäck classification into two categories: those that presented the classification correctly (in whole or in part) in the text, and those in which the scale was incorrectly reported. Publications were also divided, with respect to references, into two categories: those in which the classification was correctly cited and those in which it was cited incorrectly.

Finally, we sought to identify how many subsequent publications used as references the articles in which the classification and citation were incorrectly described. This screening, as well as the search for articles citing publications with imprecise grading and references, was initially performed and re-verified on two additional occasions by the senior author (JCG). The review of excluded publications, categorization of included articles, and results of the search for publications referencing articles with inaccurate classification and citation were sent to two independent authors (ISN and LAC) for validation, review, and suggestions for modifications. In case of disagreement, the final decision was made jointly by all three authors (ISN, LAC, and JCG).

## RESULTS

Our search identified a total of 267 articles, in addition to the two original publications by Ahlbäck^2^ and Ahlbäck et al.^3^ The following were excluded: five publications that could not be obtained in full, even after attempts through libraries and by contacting the authors via email; 34 articles published in languages other than English (10 in Chinese, seven in French, five in German, five in Turkish, two in Polish, two in Spanish, one in Portuguese, one in Croatian, and one in Lithuanian); 40 articles consisting of conference or congress abstracts; eight publications whose topic was not related to osteoarthritis (six on osteonecrosis, one on patella alta, and one on scintigraphy for evaluation of the femoral condyles); 57 articles in which the Ahlbäck classification was not found in the references; and 58 publications that did not present the Ahlbäck classification in whole or in part in the text, or that only mentioned it without adequate description.

**Figure 2:**
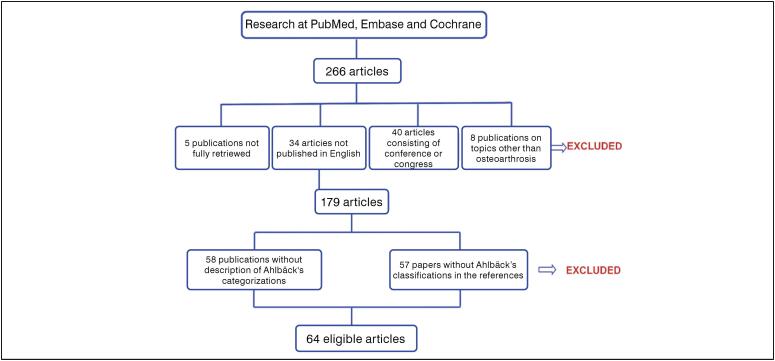
Summary of the systematic review methodology.

Thus, 64 articles were included in the analysis, in addition to the two original publications. (Figure 2)

Of the 64 articles analyzed, only 24 (36.9%) presented the Ahlbäck classification correctly. Six publications^5,14-18^ used the 1968 article,^2^ 17 articles^19-35^ used the 1980^3^ article and, one article used both scales^4^. On the other hand, 40 articles (62.5%) reported the classification incorrectly. Six of them included grade 0,^6,11,36-39^ four included grade 6,^40-43^ both of which do not exist in the original scale; 10 reported the scale in a way very different from the original description^44-53^;and 20 described grade 4 as attrition between 5 and 10 mm and grade 5 as attrition greater than 10 mm.^1, 7-10, 54-68^

With regard to citation of the reference, in 13 articles (20.3%) it was done correctly. Six of them^5,14-18^ cited the 1968 publication^2^, five,^29,30,46,52,63^ cited the 1980 publication^3^ and two,^4,31^ cited both articles. However, only two of the seven articles that adequately mentioned the 1980 publication cited it perfectly, including page 2096.^30,52^ Conversely, 51 (79.6%) publications cited it incorrectly.^1,6-11,19-28,32-45,47-51,53-62,64-68^

We found 10 articles (15.6%) in which both the classification and citation were correctly reported^4,5,14-18,29-31^ and, in 37 (58.4%), both classification and citation were incorrectly described.^1,7-11,21,36-45,47-51,53-68^ The information contained in the two paragraphs above is summarized in Table 2. Finally, we identified 766 publications that used as references articles in which the classification and citation were inaccurately described.

**Table 2:** Comparison of published information on the Ahlbäck classification and its citations in the literature, with respective authors and year of publication.

Authors	Year of publication	Correct classification	Incorrect classification	Correct citation	Incorrect citation
Lindberg & Montgomery^5^	1987	•		•	
Lysholm et al.^40^	1987		•		•
Bert et al.^41^	1989		•		•
Barrett et al.^14^	1990	•		•	
Keyes et al.^54^	1992		•		•
Rockborn et al.^19^	1996	•			•
Petersson et al.^20^	1997	•			•
Sahlström et al.^6^	1997	•			•
Petersson et al.^55^	1997		•		•
Sahlström et al.^21^	1997		•		•
Larsson et al.^22^	1998	•			•
Gillquist & Messner^42^	1999		•		•
Davies et al.^15^	1999	•		•	
Gidwani et al.^44^	2003		•		•
Hung et al.^45^	2003		•		•
Galli et al.^8^	2003		•		•
Rademakers et al.^46^	2004		•	•	
Tang et al.^23^	2004	•			•
Tang et al.^24^	2005	•			•
Sisto & Mitchell ^16^	2005	•		•	
Weidow et al.^4^	2006	•		•	
Sisto & Sarin ^17^	2006	•		•	
Kijowski et al.^36^	2006		•		•
Hing et al.^18^	2007	•		•	
Rademakers et al.^47^	2007		•		•
Beard et al.^48^	2007		•		•
Becker et al.^49^	2008		•		•
Lidén et al.^25^	2008	•			•
Rademakers et al.^50^	2009		•		•
Turajane et al.^26^	2009	•			•
Ventura et al.^56^	2010		•		•
Parmaksizoğlu et al.^27^	2010	•			•
McDonnell et al.^57^	2011		•		•
Marcacci et al.^51^	2011		•		•
Brucker et al.^52^	2011		•	•	
Hernández-Vaquero et al.^10^	2012		•		•
Moon et al.^28^	2013	•			•
Staikos et al.^29^	2013	•		•	
Wright et al.^9^	2014		•		•
Waldstein et al.^37^	2014		•		•
Li et al.^30^	2015	•		•	
Garrido et al.^31^	2015	•		•	
Ghinelli et al.^38^	2016		•		•
Martins et al.^58^	2016		•		•
Talic-Tanovic et al.^32^	2017	•			•
Skou et al.^33^	2017	•			•
Köse et al.^11^	2018		•		•
Belk et al.^59^	2018		•		•
Elveos et al.^60^	2018		•		•
Kinsey et al.^43^	2018		•		•
Lim et al.^61^	2019		•		•
Keenan et al.^1^	2020		•		•
Identeg et al.^53^	2020		•		•
Albergo et al.^39^	2020		•		•
Wing et al.^62^	2021		•		•
Pedersen et al.^63^	2021		•	•	
Jarecki et al.^64^	2021		•		•
Eckersley et al.^65^	2021		•		•
Zambianchi et al.^66^	2021		•		•
Jarecki et al.^67^	2022		•		•
Nakayama et al.^7^	2023		•		•
Obara et al.^34^	2023	•			•
Törnblom et al.^35^	2024	•			•
Schippers et al.^68^	2024		•		•
		1	2	3	4
		24	40	13	51
		37.50%	62.50%	20.31%	79.69%

## DISCUSSION

The main finding of this study was that the number of articles that correctly described the Ahlbäck classification and those that used exact citation in the references, with precise information on the publication journal, is small (36.9% and 20.3%, respectively). On the other hand, only 10 of the articles in our selection (15.6%) correctly described both the classification and citation, whereas in 37 of them (58.4%), both the classification and the citation were incorrect.

Among the 64 articles included in our review, 24 (36.9%) correctly described the classifications. Of these, six publications (25%)^5,14-18^ used the 1968 classification,^2^ articles (70.8%)^19-35^ used the 1980 classification^3^ and one publication (4.16%) used both scales.^4^ On the other hand, 40 publications (62.5%) described the classification incorrectly. The inaccuracies identified were: inclusion of grade 0, absent in the original scale, in six articles (15%),^6,11,36-39^ inclusion of grade 6, also absent in the original scale, in four publications (10%),^40-43^ description of the classification in a way significantly different from the original in 10 articles (25%),^44-53^ and misinterpretation of grades 4 and 5, in which grade 4 was described as attrition between 5 and 10 mm and grade 5 as attrition greater than 10 mm, in 20 publications (50%).^1,7-10,54-68^

While we did not identify a probable explanation for the first three inaccuracies, the last error likely resulted from confusion between the description of bone defects presented in Ahlbäck's 1968 monograph^2^ and the classification developed by Ahlbäck et al. in 1980.^3^ In the 1968 monograph^2^, Ahlbäck described bone defects as being smaller than 5 mm, between 5 and 10 mm, and greater than 10 mm. In the 1980 classification,^3^ however, grade 3 corresponds to minor bone attrition (<0.5 cm), grade 4 to moderate attrition (between 0.5 and 1.5 cm), and grade 5 to severe attrition (>1.5 cm).

Confusing this information may lead to an erroneous assessment of osteoarthritis classification and, consequently, to inappropriate therapeutic decisions if they are based on joint attrition grading. For example, a patient with 12 mm of bone attrition should be classified as grade 4 and not grade 5, according to the 1980 classification.^3^ The lack of uniformity in osteoarthritis classifications may hinder treatment indication as well as the interpretation and comparison of results across different authors, especially when these are based on gonarthrosis grades assessed by weight-bearing anteroposterior radiographs.

Regarding bibliographic references, only 13 publications cited the original sources correctly. Among them, six articles^5,14-18^ correctly mentioned the 1968 publication^2^, five^29,30,46,52,63^ appropriately cited the 1980 publication^3^ and, in two publications both references were cited.^4,31^ However, only two of these seven articles (28.5%) in which the Ahlbäck classification was correctly used inserted the reference to the specific page (page 2096).^30,52^

This failure can be explained by a detail in the Ahlbäck et al. publication.^3^ The original article, published in Läkartidningen, begins on page 2091 and appears to end on page 2093. After that page, there is an intercalated publication, which may give the impression that the article had ended. However, the publication resumes on page 2096, where the complete classification is described. This layout of the journal may have induced citation errors.

In 51 articles, the bibliographic references did not correspond to the classification described in the text.^1,6-11,19-28,32-45,47-51,53-62,64^ Among these, nine^11,23,24,26,28,34,36,38,39^ accurately reported the 1980 classification^3^ in the text of the publication but inexplicably cited the 1968 classification.^2^ Surprisingly, classification and citation were both correctly reported in only 10 articles (15.6%)^4,5,14-18,29-31^ whereas in 37 publications (58.4%), both classification and citation were incorrectly described.^1,7-11,21,36-45,47-51,53-68^

We identified 766 publications that used as references the 37 articles in which the classification and citation were inaccurately described. This represents an average of more than 20.7 citations per erroneous article, demonstrating a significant multiplier effect. Such propagation of errors may contribute to the dissemination of bias and generate methodological inconsistencies in the literature, compromising standardization, reproducibility, and comparability of results in subsequent studies.

In summary, for the appropriate use of the Ahlbäck knee osteoarthritis grading classification on weight-bearing anteroposterior radiographs, it is recommended to use the Ahlbäck et a.^3^ publication as the reference, since it contains the description of the knee osteoarthritis grading according to joint attrition. Authors should cite the original sources correctly, including page 2096 in the reference to the 1980 publication^3^ and carefully review the description of the classification grades, avoiding adaptations or modifications not grounded in the original literature, as these may impact therapeutic approaches, particularly in the evaluation of outcomes.

Our study has some limitations. It was not possible to obtain all publications identified in the search, even after library requests and direct attempts to contact the authors by email. Furthermore, we excluded articles in which only the osteoarthritis categorization was described but not cited in the bibliography, and vice versa. We also did not perform comparisons between the Ahlbäck classification and other classifications used internationally, nor those including lateral radiographs, since our aim was solely to compare the original texts of Ahlbäck's 1968 article,^2^ and the 1980 publication by Ahlbäck et al.^3^, with what was written in subsequent works that used the classifications and cited them in their references. The clinical relevance of this study lies in emphasizing the importance of correctly using the Ahlbäck classification, aiming at standardization of therapeutic decisions and accurate evaluation of outcomes.

## CONCLUSION

Although the Ahlbäck classification continues to be used for radiographic assessment of knee osteoarthritis, only 10 of the articles in our systematic review (15.6%) correctly described both the classification and its citation, whereas in 37 of them (58.4%), both were incorrectly reported. The latter were cited as references in 766 publications, which may have contributed to the dissemination of bias and methodological inconsistencies in the scientific literature.
